# Suicidal risk in Latino patients with schizophrenia

**DOI:** 10.1192/j.eurpsy.2023.2296

**Published:** 2023-07-19

**Authors:** O. Olive, J. A. Ontiveros, A. A. Perez, M. F. Serna

**Affiliations:** Psychiatry, University Hospital “Dr José E. González” Monterrey, Nuevo Leon, Mexico, Monterrey, Mexico

## Abstract

**Introduction:**

Schizophrenia is a highly debilitating disorder afflicting more than 24 million individuals worldwide. In Mexico, the Ministry of Health estimates that it affects more than 1 million people. Suicide is one of the main causes of death among people diagnosed with schizophrenia, their risk is 12 times higher than in. the general population

**Objectives:**

To evaluate the clinical characteristics of schizophrenic patients at risk of suicide in the Latino population.

**Methods:**

We included 130 patients recruited from genetics studies in Latino patients with schizophrenia from the outpatient and inpatient psychiatric ward of the University Hospital “Dr José E. González” in Monterrey, Nuevo Leon, Mexico. Beck Depression Inventory (BDI-II), the Convergent Functional Information for Suicidality (CFI-S) were applied to all participants. We compared the sociodemographic and clinical characteristics of patients with suicidal risk (measured by history of suicidal attempt or current suicidal risk) and present depressive episode.

**Results:**

Of the 130 participants, 66.9% were male, the median age was 38 years. We found 11(14.3%) patients with suicidal risk and 119 (91.5%) without suicidal risk. Sociodemographic and clinical characteristics of the study population at risk of suicide are described in graphic 1. Patients with a history of suicide attempt scored higher on the CFI-S scale with a median of 0.5 (q1=0.45; q3=0.54) vs. 0.31 (q1=0.22; q3=0.45) (p= 0.004)(Graphic 2). Based on the BDI-II we found 2.30% patients showed a mild depression, 20.0% moderate depression and 4.61% severe depression (graphic 3). Schizophrenic patients with a previous suicide attempt and depressive episode had higher score range in CFI-S, median .65 (q1=.65; q2=.59, p=0.000). Also, 63.60% were severely depressive (p=0.000) when they compared with patients with low risk of suicide. Schizophrenic patients with suicidal risk were characterized by: age >=60 years old, unemployment, no children, single, without religion, family history of suicide, previous suicide attempt, depressive episodes, substance abuse, auditory hallucinations and referential delusions.

**Image:**

**Figure:**
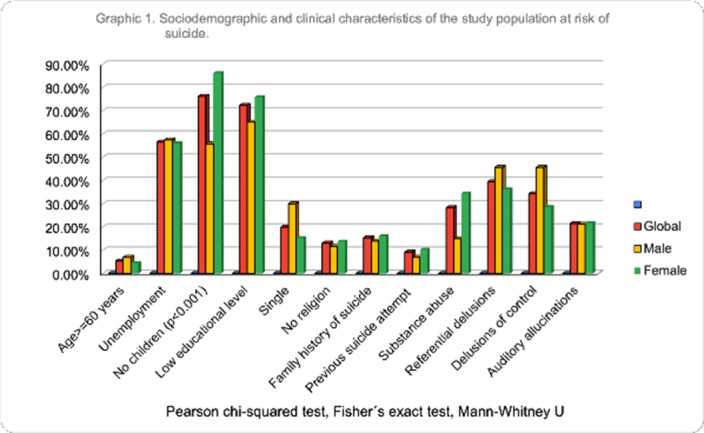

**Image 2:**

**Figure:**
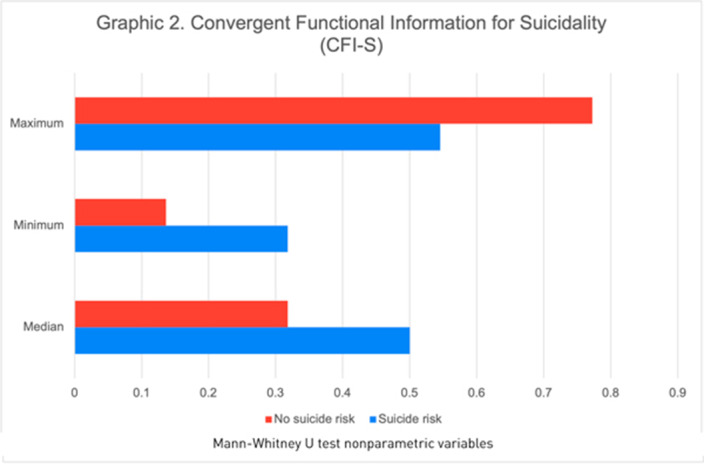

**Image 3:**

**Figure:**
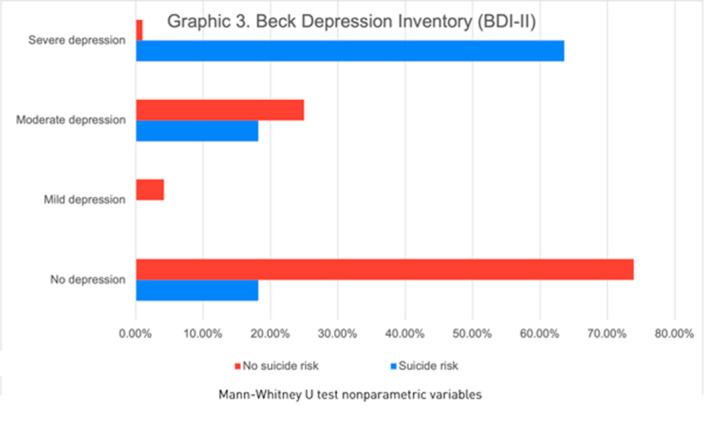

**Conclusions:**

In our study with Latino population, we observed similar clinical characteristics predictive of suicide risk described in the international studies. Our study is relevant to applied preventive measures in groups of schizophrenic patients with risk factors and their relatives.

**Disclosure of Interest:**

None Declared

